# 3D Synthetic Peptide-based Architectures for the Engineering of the Enteric Nervous System

**DOI:** 10.1038/s41598-019-42071-7

**Published:** 2019-04-03

**Authors:** Paola Brun, Annj Zamuner, Alessandro Peretti, Jessica Conti, Grazia M. L. Messina, Giovanni Marletta, Monica Dettin

**Affiliations:** 1 Department of Molecular Medicine, University of Padova, Via Gabelli, 63, Padova, 35121 Italy; 2 Department of Industrial Engineering, University of Padova, Via Marzolo, 9, Padova, 35131 Italy; 3 Department of Chemical Sciences, University of Catania, Via A. Doria, 6, Catania, 95125 Italy

## Abstract

Damage of enteric neurons and partial or total loss of selective neuronal populations are reported in intestinal disorders including inflammatory bowel diseases and necrotizing enterocolitis. To develop three-dimensional scaffolds for enteric neurons we propose the decoration of ionic-complementary self-assembling peptide (SAP) hydrogels, namely EAK or EAbuK, with bioactive motives. Our results showed the ability of EAK in supporting neuronal cell attachment and neurite development. Therefore, EAK was covalently conjugated to: RGD, (GRGDSP)_4_K (fibronectin), FRHRNRKGY (h-vitronectin, named HVP), IKVAV (laminin), and type 1 Insulin-like Growth Factor (IGF-1). Chemoselective ligation was applied for the SAP conjugation with IGF-1 and the other longer sequences. Freshly isolated murine enteric neurons attached and grew on all functionalized EAK but IGF-1. Cell-cell contact was evident on hydrogels enriched with (GRGDSP)_4_K and HVP. Moreover (GRGDSP)_4_K significantly increased mRNA expression of neurotrophin-3 and nerve growth factor, two trophic factors supporting neuronal survival and differentiation, whereas IKVAV decoration specifically increased mRNA expression of acetylcholinesterase and choline acetyltransferase, genes involved in synaptic communication between cholinergic neurons. Thus, decorated hydrogels are proposed as injectable scaffolds to support *in loco* survival of enteric neurons, foster synaptic communication, or drive the differentiation of neuronal subtypes.

## Introduction

**Figure 1:**
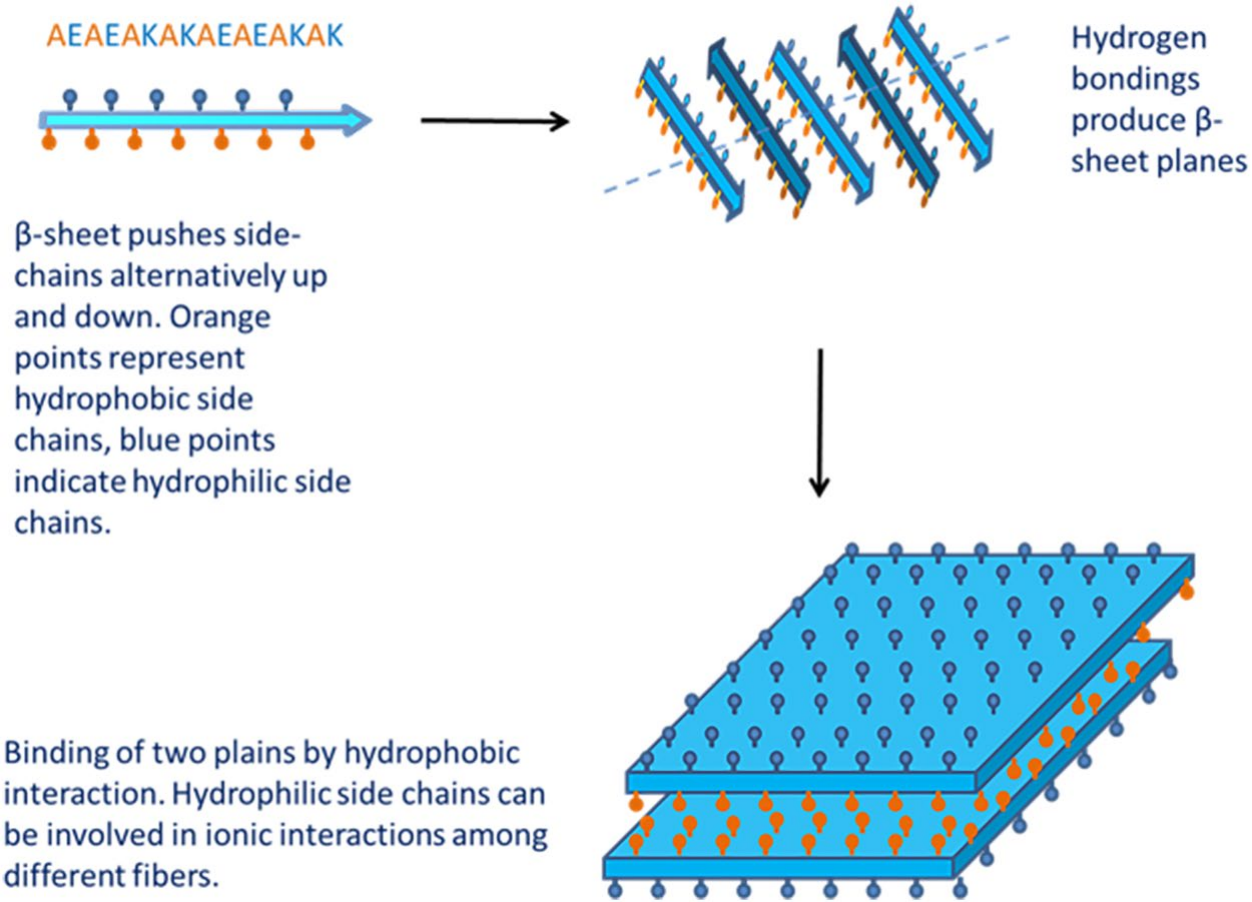
Scheme of self-assembly of peptides. At the top, left side: hydrophobic residues (orange) and hydrophilic residues (blue) alternate in the sequence of EAK peptide. The arrows indicate aggregation of EAK in β-sheet layers.

The extracellular matrix (ECM), the network of glycoproteins providing mechanical support and biochemical signals for tissue homeostasis, plays a key role in the economy of biological tissues. The ECM determines polarization and motility of cells and tunes the local concentration of adhesive proteins, growth factors, and glycosaminoglycans. Therefore, a big challenge in regenerative medicine is to design artificial ECM mimicking the *in vivo* 3-dimensional (3D) architecture of fibrous proteins, the nano- and micro-scale structural complexity, the tensional cues, and the interaction with the cytoskeletal network of the cells to eventually support a more dynamic environment for cell motility, wound healing and tissue reconstruction^1^. By now, traditional two-dimensional cell culture systems have been overcome by 3D scaffolds that more closely reproduce the *in vivo* architecture of the tissue^2,3^. In this scenario, nanofibrous structure and self-assembling materials gained considerable interest as scaffolds able to properly control cell-ECM interactions and *in loco* activity of bioactive factors. Scaffolds of self-assembling peptides (SAPs) were described at first by Zhang in 1992 who reported Zoutin protein fragments assuming highly stable β-sheet conformation in solution. However, SAPs are naturally occurring structures usually generated by simple molecules organized in nano- or micro-metric structures by means of weak interactions such as hydrogen or Van der Waals bonds^4,5^. In SAP scaffolds, hydrophilic (positively or negatively charged) and hydrophobic residues alternate resulting in amphiphilic β-sheet layers that in turn stack one with the other (Fig. 1).

The resulting scaffolds are hydrogels made of water (99% of the composition) and woven nanofibers with mean length ranging from hundreds of nanometers to few micrometers^6^. The network of SAP fibers supports the exchange of bioactive factors, oxygen, nutrients, and waste products among cells and the environment, but most of all SAP hydrogel provides a framework resistant to high temperature (90 °C for 4 hours) and digestion mediated by proteolytic enzymes^4–7^. Therefore, SAP scaffolds have been proposed as suitable cell supports in tissue engineering. Moreover, features of SAPs are easily scalable since the self-assembling process can be controlled by temperature, pH, concentration and nature of positive monovalent ions. Thanks to their compatibility and versatility, SAP hydrogels have been extensively proposed for application in regenerative medicine and the most promising application concerns the engineering of nervous tissue^8–12^. Indeed, electrospun cylinders filled with SAPs were successfully used to repair chronic injuries in the spinal cord^13^ and to restore the optic tract in hamsters^14^.

**Figure 2:**
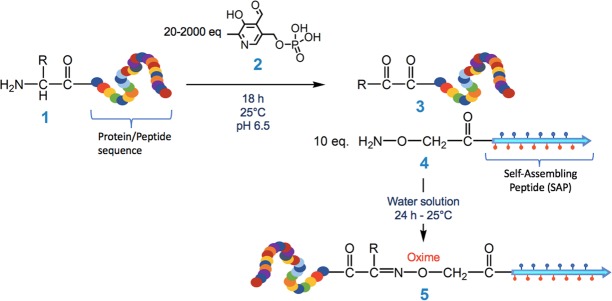
Scheme of chemoselective ligation. Step 1, PLP-driven transamination changes the N-terminal Gly in α-ketoaldehyde; step 2, reaction between α-ketoaldehyde and oxyamino group generates oxime.

SAP hydrogels have the potential to be excellent supports in regenerative medicine of the nervous system due to the nanofibrous structure, the biomechanical properties and the ability to accelerate serum protein adsorption. However, the application of SAPs is strongly jeopardized by the lack of adhesive motives able to properly drive attachment, growth, and differentiation of specific neuronal cells on hydrogels. To fill this gap, in this study we proposed the enrichment of plain SAPs with self-assembling sequences joining bioactive factors. The simple and key idea of our study was to create ECM scaffolds by blending SAP-conjugated bioactive molecules with plain SAPs. Since de-absorption of biological factors is usually involved in inhibition of adhesion^15^, we decided to covalently bind the bioactive motives to the hydrogels. However, to keep the SAP side chains available for cell receptor interaction, two strategies were adopted in covalent binding: (i) extension in the protocol of solid phase peptide synthesis to condense short bioactive fragments (≤5 amino acids) to the self-assembling sequence; (ii) chemoselective ligation for the conjugation of SAPs with longer bioactive peptides. Indeed, chemoselective ligation ensures a bio-orthogonal reaction under mild conditions and without perturbing protein tertiary structure (Fig. 2, 3 + 4 → 5) whereas transamination using pirydoxal-5-phosphate (PLP) converts the first amino acid of the peptide/protein sequence in ketone or aldehyde (Fig. 2, 1 → 3) under non-denaturing conditions, thus introducing a functional group for chemoselective ligation^16,17^.

As compared with superficial addition, conjugation of bioactive motifs with SAPs provides, through self-assembling, the decoration of the whole hydrogel volume rather than only its surface and offers the possibility to build precise microenvironments for selected cellular systems.

Hydrogels enriched with conjugates (i.e. EAK-(GRGDSP)_4_, EAK-HVP, and EAK-IGF-1) have been successfully used in our previous studies as scaffolds to induce adhesion and growth of mesenchymal stem cells^18^. Moreover, we demonstrated the advantage in adding a spacer among the bioactive sequences and the aldehyde group to optimize the chemoselective ligation protocol^19^. In the present work, we proposed the decoration of 3D self-assembling matrices with new adhesive motives and previously reported conjugates^18^ in different combinations to provide scaffolds for the engineering of the enteric nervous system (ENS). The ENS is a complex network of glial cells and motor and sensory neurons embedded in the gastrointestinal wall that controls motility, secretion, digestion, and even pain perception and inflammatory process of the gut^20^. Enteric neuropathies are commonly associated with loss of enteric neurons and functional gut disorders ranging from inflammatory bowel diseases to chronic constipation and necrotizing enterocolitis^21^.

To support the *in vitro* growth of enteric neurons, in this study ionic self-assembling peptides of module II (EAK or EAbuK)^2^ were conjugated with (i) RGD motif; (ii) linear peptide of 4 GRGDSP motives previously reported to support adhesion in osteoblasts, cardiomyocytes and endothelial cells^15,22,23^; (iii) (351–359) fragment of the human vitronectin involved in proteoglycan-mediated osteoblast adhesion^22,24,25^; (iv) laminin sequence IKVAV^26^; (v) insulin-like growth factor-1 (IGF-1) promoting survival of cortical neurons^27^. Freshly isolated murine enteric neurons populated SAP hydrogels and modulated gene expression in response to specific scaffold enrichment. Up today, peptide nanofiber hydrogels have been used for regeneration of the central nervous system^10^. Our work demonstrated that hydrogels can be successfully applied also in recovery of injured enteric nerves. Moreover, since phenotype of enteric neurons is strictly connected to ECM composition^28^, data reported in the present study further confirmed that designing scaffolds for the engineering of the enteric nervous system is feasible and crucial in tissue regeneration during severe clinical conditions such as megacolon, short bowel syndrome, or inflammatory bowel disease^21^. Knowledge about the influence of the ECM composition in neuronal stem cell differentiation proved that enteric cell transplantation in aganglionic gut areas has therapeutic effects^29–31^. However, our data demonstrated that application of injectable hydrogel matrices decorated with different motives could support gut neuronal plasticity possibly fostering *in loco* migration and differentiation of definite intestinal neuronal subtypes but avoiding inoculation of foreign biological materials.

## Results and Discussion

### CD analysis of peptides in PBS solution

**Figure 3:**
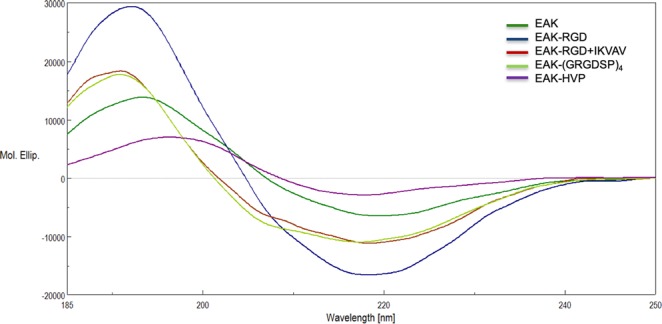
CD spectra of EAK (dark green), EAK-RGD (blue), EAK-RGD + IKVAV (red), EAK-(GRGDSP)_4_ (light green) and EAK-HVP (violet). CD analyses were carried out in 10 mM phosphate buffer pH 7 with 5 × 10^−5^ M peptide concentration.

As reported in Fig. 3, the typical β-sheet spectra were observed for all EAK-based peptides in PBS solution, indicating the integrity of principal secondary structure was not perturbed by the addition of adhesion cues. In fact, the positive band remains around 194 nm (the spectrum of EAK is reported for comparison^32^), whereas the negative band is around 217 nm. Considering the EAK-HVP spectrum, the red shift of positive band and the reduction of its intensity with respect to EAK one, might result from the contribution of HVP conformation (type II β-turn) that is characterized by a negative band at 190 nm (Supplementary Information, Fig. S1). The spectrum of EAbuK, published in Gambaretto R. *et al*. confirmed a predominant β-sheet structure^32^.

### Structure of EAK, EAbuK and EAK enriched with bioactive sequences

**Figure 4:**
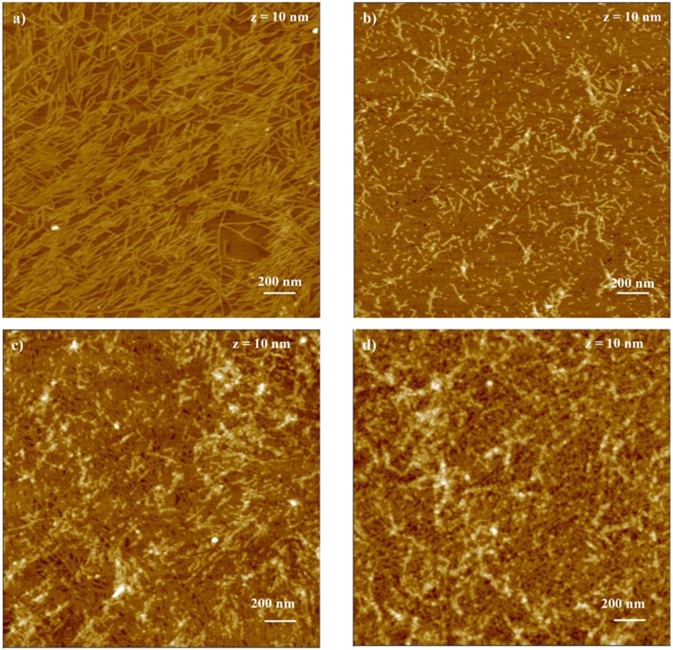
AFM height images of: (**a**) EAK, (**b**) EAbuK, (**c**) EAK-RGD, (**d**) EAK-RGD + IKVAV. The concentration for all samples was 0.15% w/v. One hour of adsorption on mica surface.

Figure 4 reports the atomic force microscopy images of a) EAK, b) EAbuK, c) EAK-RGD (EAK: EAK-RGD, 1:1) and d) EAK-RGD + IKVAV (EAK: EAK-RGD + IKVAV, 1:1) peptides (0.15% w/v) on freshly cleaved mica surfaces, after 1 hour of incubation in buffer solution. It can be seen that EAK sequences yield a remarkable self-organization process already after an hour of incubation, i.e. at very short time with respect to the employed cell incubation time, producing a relatively dense network of mostly parallel aligned nanofibrils.

The characteristic dimensions of the self-assembled fibrils have been determined by applying the Canet-Ferrer method that takes into account the tip-fibrils convolution effects arising from the finite dimensions of the AFM tip^33^. This model yields the effective corrected W_eff_ width based on the measured fibrils width. In the present case, the average fibril width for EAK peptide, is 5.3 ± 0.5 nm and height is 1.2 ± 0.4 nm, while the average length is variable in a range of 140.0–630.0 nm. These data are in agreement with the previous results, i.e., W_eff_ = 5.9 ± 1.1 nm for the corrected width (assuming a flat configuration of the sequences) and h = 1.1 ± 0.1 nm for the height value^34^. Therefore, in this case, with respect to previous results obtained at very low concentration (∼10^−6^ M) and with LiCl in the buffer, a much higher density and related higher bifurcation events of the assembled fibrils are found, due to the striking difference in EAK concentration and the nature of the buffer solution employed (containing no Li salt).

Contrarily, EAbuK sequences, differing only for the presence of an ethyl group as side chain of Abu instead of a methyl group as side chain of Ala, don’t form a significant amount of fibrils, giving mostly very short fibers peptide aggregates, with a typical height of 1.7 ± 0.2 nm and the effective width of 7.6 ± 1.1 nm. In particular, Fig. 4b shows small nanometric holes suggesting the presence of a peptide monolayer formed on mica substrate. The peptide carpet formation is likely due to the strong electrostatic interactions between peptide and surface. The peptide monolayer thickness measured by AFM section analysis is 1.2 ± 0.01 nm and it suggests a side-on molecules arrangement.

The enrichment of EAK sequence with EAK-RGD (Fig. 4c) produces the formation of a two-level system, an underlayer consisting of a compact network with fibrils 1.5 ± 1.1 nm thick and high cross-linking density, and an overlayer of sparse and short fibers aggregates (average hight = 2.1 ± 1.4 nm). The fibrils and the fibrous aggregates of the two layers have roughly the same width, about 12.1 ± 1.1 nm.

The presence of IKVAV decoration on the EAK-RGD + IKVAV hydrogel (Fig. 4d) causes the formation of a first layer with a less dense network, with narrower fibrils (width = 9.8 ± 0.7) and larger meshes and an increase of the fibrillar aggregation of the second layer characterized by a bigger width (17.9 ± 1.4 nm).

The AFM analysis highlights the formation of the fibrils only in the presence of EAK that should be attributed to the ß-sheet secondary peptide folding where the hydrophobic alanine units are thought to promote the coupling of the hydrophobic sides of the two adjacent molecules, coupled with the electrostatically-driven peptide-peptide interaction involving the oppositely charged lysine and glutamic acid residues on the two adjacent sequences, yielding the ordered growth of the fibrils in a checkerboard-like manner.

On the other hand, the unstructured nature of the EAbuK aggregates could not be explained at this stage of the study, because EAbuK has the same length of EAK peptide. This may suggest that the presence of the side chain of amino butyric acid, hinders efficiently the self-organization process.

The fibrillary self-organization process of EAK has been shown previously to boost the cell adhesion and proliferation, in agreement with the present results, while EAbuK unstructured aggregates appear to be significantly less effective in promoting cell adhesion processes. Accordingly, we attribute to the ordered arrangement of the peptides the capability of efficiently interacting with the cell adhesion “mechanism”.

### Biological characterization

**Figure 5:**
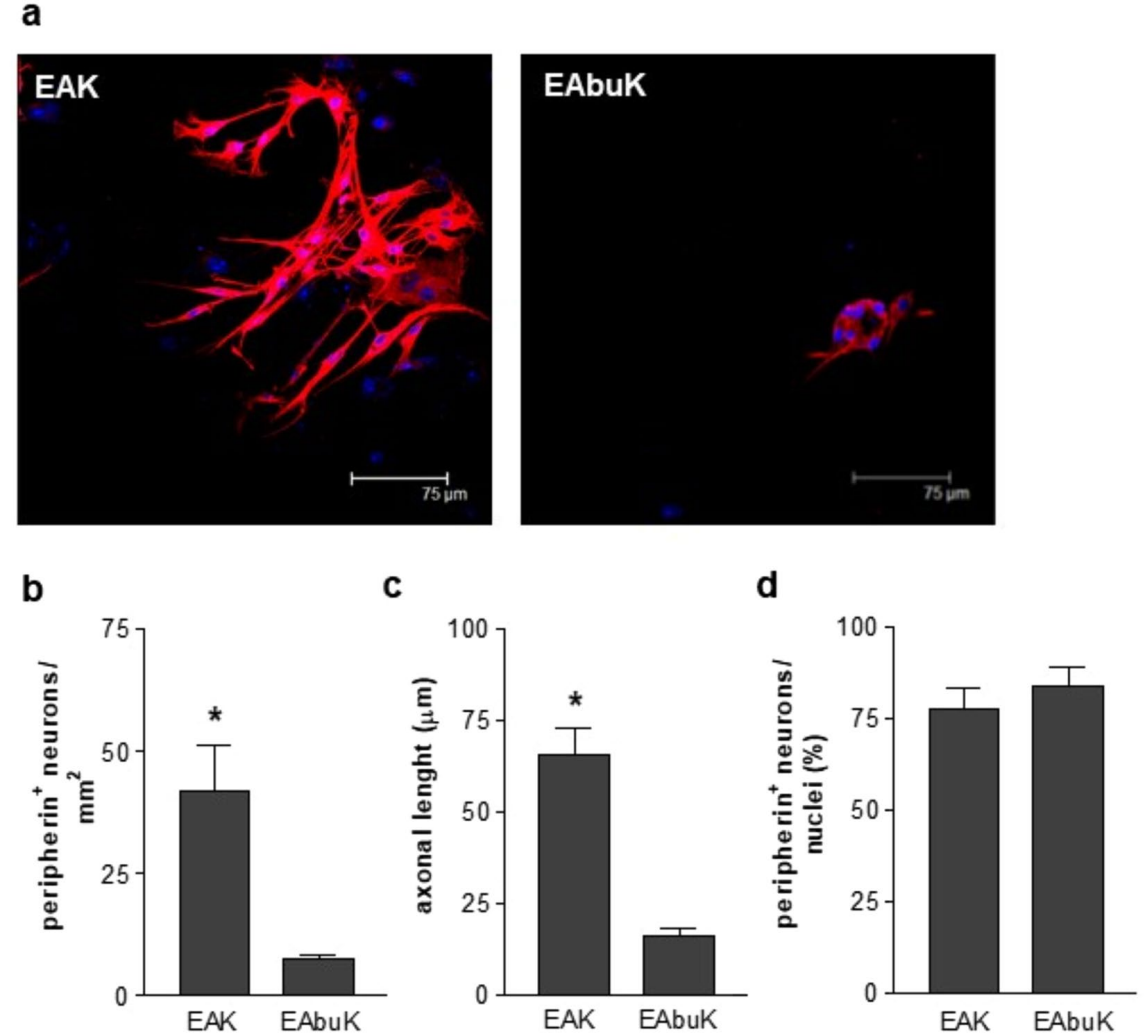
Enteric neurons cultured on EAK and EAbuK (0.15% w/v). (**a**) Immunofluorescent analysis of cells cultured for 6 days and labeled with anti-peripherin antibody (red). Nuclei were stained with TOTO-3 iodide (blue). (**b**) Mean number of peripherin positive cells. *Denotes *p* < 0.001. (**c**) The axonal length was calculated using ImageJ. *Denotes *p* < 0.001. (**d**) Percentage of peripherin positive cells on total nuclei.

In order to evaluate the biological properties of SAPs, EAK and EAbuK were deposited on sterile glass coverslips (0.15% w/v on 1 cm^2^) and incubated for 1 hour. Freshly isolated murine enteric neurons were seeded on glass coverslips coated with SAPs and cultured for 6 days. As reported in Fig. 5a, cells were seeded on EAK and stained with anti-peripherin antibody, a marker of neuronal cells, developed a frank neuronal phenotype characterized by organization in ganglia and several neurites extending from the soma to neighbouring neurons^35^. On the contrary, EAbuK did not support adhesion of neurons neither extension of neurites. Indeed, cells cultured on EAbuK were barely positive for neuronal peripherin marker and showed rounded shape with small or absent projections (Fig. 5b).

Indeed, quantitative analysis of confocal images reported lower number of peripherin positive cells and shorter axonal networks in EAbuK with respect to cells cultured in EAK (*p* < 0.001; Fig. 5b,c). Overall, EAbuK did not report a strong ability in adhesion of neurons (Fig. 5a) but all attached cells were peripherin positive (Fig. 5d). By matching the biological results with AFM structural characterization of SAPs (Fig. 4) we infer that the self-assembling process of EAK resulting in a dense organized network of parallel fibrils supports adhesion and proliferation of neuronal cells. On the contrary, unstructured aggregates described in EAbuK (Fig. 4a) significantly impairs cell adhesion and phenotype development. Indeed, during morphogenesis organization of extracellular fibrils in precise geometric structures is mandatory for cell dynamic interaction^36^. At the same, organization of tenascin in parallel fibrils is required for integrin-mediated neuronal migration and development of the cerebellum^37^.

**Figure 6:**
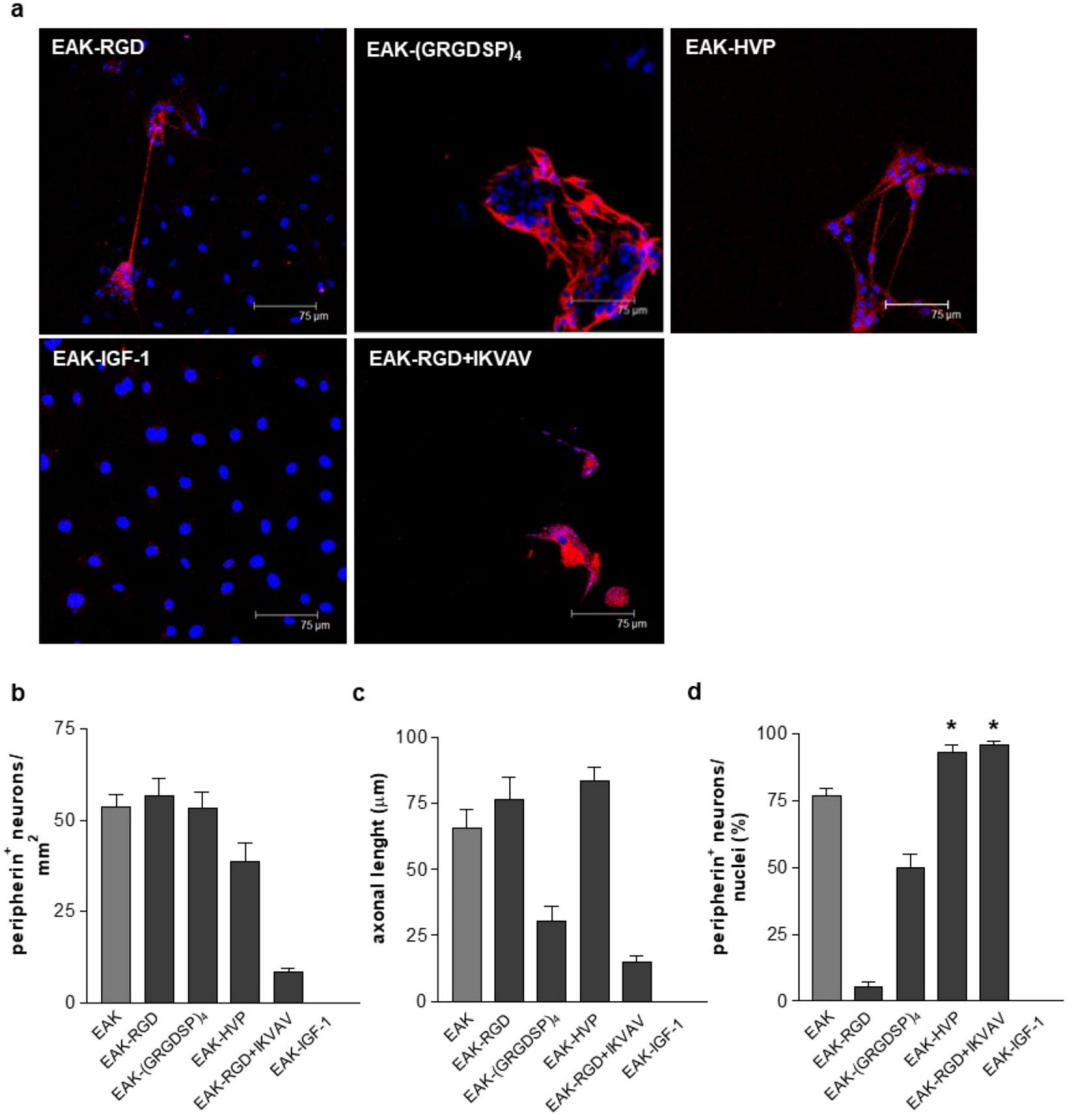
Enteric neurons growth on conjugated EAK. (**a**) Immunofluorescent analysis of neurons cultured for 6 days and labeled with anti-peripherin antibody (red). Nuclei were stained with TOTO-3 iodide (blue). (**b**) Mean number of peripherin positive cells. (**c**) The axonal length was calculated using ImageJ. (**d**) Percentage of peripherin positive cells on total nuclei. *Denotes *p* < 0.05 vs EAK.

Taking into consideration the above described results, subsequent biological analysis were performed on EAK hydrogels enriched with bioactive motives (namely RGD, (GRGDSP)_4_, HVP, IGF-1, or RGD + IKVAV) deemed to foster neuronal adhesion and differentiation^22–27^. As reported in Fig. 6, enrichment of EAK differentially supported neuronal attachment and growth as well as the outgrowth of neuronal projections.

When compared with non enriched EAK, functional motives did not improve adhesion and axonal outgrowth of neuronal cells (Fig. 6b,c). Indeed, the number of peripherin positive cells was comparable in EAK-RGD, EAK-(GRGDSP)_4_, and EAK-HVP and even decreased when cells were cultured on EAK-RGD + IKVAV and EAK-IGF-1 where peripherin negative cells were costantly observed in 5 different experiments. As compared to non functionalized EAK, EAK-HVP and EAK-RGD + IKVAV increased the selectivity for attachment and growth of peripherin positive neuronal cells (Fig. 6d, p < 0.05). Indeed, 95.0 ± 4,9% of cells cultured on EAK-HVP and 97.4 ± 2.5% of cells grown on EAK-RGD + IKVAV positively stained for peripherin as compared to 76.4 ± 6.2% of cells on EAK. Only 59.4 ± 4.8% of cells cultured on EAK-(GRGDSP)_4_ stained with anti-peripherin antibody (Fig. 5a,d). Nevertheless, HVP functionalization preserved the ability observed in non functionalized EAK in inducing neurite outgrowth (Fig. 6c).

We previously detailed the hydrogel morphology at the nanoscale^18^. AFM analysis demonstrated that addition of active motives to SAP backbone did not affect nanofiber structures that preserved features similar to those described in pure SAP. On the contrary, the main effect of conjugates addition was the inhibition of long fiber formation, observed in plain SAP. In addition, rheological analysis (small amplitude oscillatory shear tests) was carried out on EAK and IGF-1-conjugate-enriched EAK hydrogels and reported that IGF-1 conjugates did not alter the viscoelastic properties of the hydrogels^18^. Thus, the lack of bioactivity in EAK-IGF-1 conjugates described in the present study cannot be explained by the alteration in the biomechanical properties of the hydrogel but rather by the binding with the SAP and the consequent poor accessibility of the IGF-1 N-terminus to the cellular receptors. Indeed, several published data underlying the importance of IGF-1 N-terminal residues for the biological activity, partially explain our results. Indeed, peptides lacking five or six residues at the N-terminal part of IGF-1 weakly interact with cell receptors and fail in reporting biological effects^38,39^.

**Figure 7:**
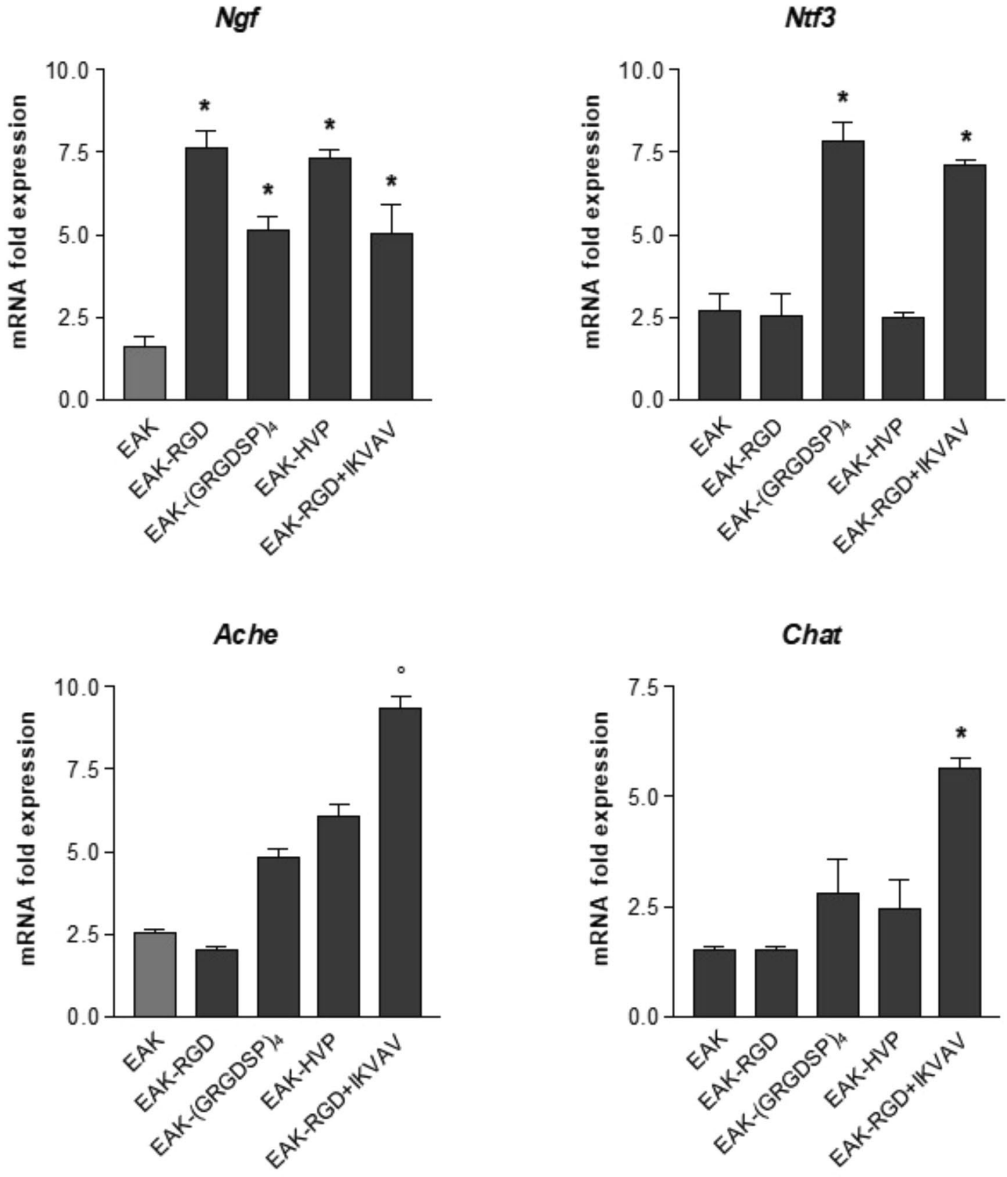
mRNA transcript levels measured by quantitative PCR in enteric neurons grown for 6 days differently functionalized EAK. *Ntf3*: Neurotrophin 3; *Ngf*: Nerve growth factor; *Ache*: Acetylcholinesterase; *Chat*: Choline acetyltransferase. Data are reported as fold change over mRNA levels determined in cells seeded and growth on glass coverslips. *Denotes *p* < 0.001; °denotes *p* < 0.05 *vs* cells cultured on EAK.

While enrichment of EAK with functional motives did not improve neuronal attachment and differentiation, RGD, (GRGDSP)_4_, HVP, and RGD + IKVAV reported different ability in modulating mRNA transcript levels (Fig. 7). Indeed, all the tested bioactive factors increased expression of mRNA specific for nerve growth factor (*Ngf*), a soluble factor involved in survival and differentiation of motor and sensory neurons^40^. Even if RGD + IKVAV decoration did not sustain the overall growth and differentiation of peripherin positive cells (Fig. 6), it increased neurotrophin 3 (*Ntf3*) mRNA transcript levels at the same extent of (GRGDSP)_4_ (Fig. 7). Moreover, among the different functional motives tested in this study, RGD + IKVAV decoration was the only one able to increase mRNA transcript levels specific for acetylcholinesterase (*Ache*) and choline acetyltransferase (*Chat*), genes involved in synaptic communications between cholinergic motor neurons (Fig. 7).

In conclusion, in this study we showed that SAP conjugated with different bioactive molecules are promising options for enteric nerve regeneration. Indeed, self-assembling hydrogels offer the opportunity to be injected or dabbed onto intestinal areas affected by loss of neurons, a histological hallmark usually associated with gastrointestinal dysfunctions^41^. Moreover, as we demonstrated in this study, specific decoration of SAP promotes different gene expression in neuronal cells thus sustaining specific functional recovery. The use of self-aggregating peptides provides an easy strategy to design and realize 3D matrices decorated with controlled concentrations of adhesion and growth factors. These molecular architectures have demonstrated great potential in the promotion of enteric nerve regeneration. In our study, different decorations of SAPs, except for IGF-1 conjugate, induced unique gene expression and neuronal cell phenotype. These preliminary results pave the way to targeted research supporting survival of neurons, fostering synaptic communication and driving neuronal subtypes differentiations. The selection and stimulation of certain type of neurons using injectable SAP scaffolds are of promising benefit for the treatment of gastrointestinal disorders caused by the lack of specific neuronal subpopulations.

## Materials and Methods

### Materials

The solid support, Rink Amide MBHA resin, was purchased from Novabiochem (Merck KGaA, Darmstadt, Germany). The Fmoc protected amino acids were provided by Novabiochem. The coupling reagents 2-(1H-Benzotriazole-1-yl)-1,1,3,3-tetramethyluronium hexafluorophosphate (HBTU), 1-[Bis(dimethylamino)methylene]-1H-1,2,3-triazolo[4,5-b]pyridinium 3-oxid hexafluorophosphate (HATU), 1-Hydroxybenzotriazole (HOBt) and 3H-[1,2,3]-Triazolo[4,5-b]pyridin-3-ol (HOAt) were supplied by Advanced Biotech (Seveso, MI, Italy). *N*,*N*-diisopropylethylamine (DIEA) and piperidine were purchased from Biosolve (Leenderweg, Valkenswaard, The Netherlands). 2,4,6-Collidine was from Janssen Chimica NV (Beerse, Belgium). Triethoxysilane (TES) and pyridoxal-5-phosphate (PLP) were from Sigma-Aldrich (Steinheim, Germany). Solvents such as *N*,*N*-dimethylformamide (DMF), trifluoroacetic acid (TFA), *N*-methyl-2-pyrrolidone (NMP) and dichloromethane (DCM) were from Biosolve. Increlex (10 mg/mL IGF-1 solution) was from Ipsen Pharma (Boulogne-Billancourt, France).

### Peptide Synthesis

#### EAK

The detailed solid phase peptide synthesis, purification and characterization of EAK (H-Ala-Glu-Ala-Glu-Ala-Lys-Ala-Lys-Ala-Glu-Ala-Glu-Ala-Lys-Ala-Lys-NH_2_) are reported in Zamuner *et al*.^18^. Final purity grade 99.2%. The analytical HPLC chromatogram of purified peptide is reported in Supplementary Information (Fig. S2).

#### Aoa-EAK

The Aoa-EAK peptide, sequence: NH_2_-O-CH_2_-CO-Ala-Glu-Ala-Glu-Ala-Lys-Ala-Lys-Ala-Glu-Ala-Glu-Ala-Lys-Ala-Lys-NH_2_, was synthesized as reported elsewhere^18^.

#### G7(GRGDSP)_4_K

The peptide G7(GRGDSP)_4_K (sequence: H-Gly-7-aminoheptanoic acid-(Gly-Arg-Gly-Asp-Ser-Pro)_4_-Lys-NH_2_) was synthesized, purified and characterized as reported in Zamuner *et al*.^18^.

#### α-Ketoaldehyde-7-Aminoheptanoicacid-(GRGDSP)_4_K

The conversion of G7(GRGDSP)_4_K in α-ketoaldehyde-7-aminoheptanoic acid-(GRGDSP)_4_K (sequence: H-CO-CO-NH-(CH_2_)_6_-CO-(Gly-Arg-Gly-Asp-Ser-Pro)_4_Lys-NH_2_) was obtained by addition of 23.63 mg of G7(GRGDSP)_4_K (9.061 × 10^−6^ moles) to 18.12 mL of 10 mM pyridoxal-5-phosphate (PLP) in 25 mM sodium phosphate buffer pH 6.5 (181.23 × 10^−6^ moles PLP; G7(GRGDSP)_4_K:PLP = 1:20) for 18 h at 37 °C. The α-ketoaldehyde-peptide was isolated and characterized as reported in Zamuner *et al*.^18^.

#### EAK-(GRGDSP)_4_

The chemoselective ligation between α-ketoaldehyde-7-aminoheptanoic acid-(GRGDSP)_4_K and Aoa-EAK produced the conjugate named EAK-(GRGDSP)_4_. Briefly, 25.894 × 10^−6^ moles of Aoa-EAK was added to 2.589 × 10^−6^ moles of α-ketoaldehyde-7-aminoheptanoic acid-(GRGDSP)_4_K (ratio: 10:1) in 25.89 mL of H_2_O MilliQ for 24 hrs at room temperature. The product was isolated using RP-HPLC. The identity of the product was confirmed by MALDI mass analysis^18^. The analytical HPLC chromatogram and ^1^H NMR spectrum of purified conjugate EAK-(GRGDSP)_4_ are reported in Supplementary Information (Figs S3 and S9).

#### G7HVP

The peptide G7HVP (sequence: H-Gly-7-aminoheptanoic acid-Phe-Arg-His-Arg-Asn-Arg-Lys-Gly-Lys-NH_2_) was synthesized by standard Fmoc chemistry and a fully automated peptide synthesizer. The detailed synthesis, purification and characterization are reported elsewhere^18^.

#### α-Ketoaldehyde-7-Aminoheptanoic Acid-HVP

The conversion of G7HVP in α-ketoaldehyde-7-aminoheptanoic acid*-*HVP (sequence: H-CO-CO-NH-(CH_2_)_6_-CO-(Phe-Arg-His-Arg-Asn-Arg-Lys-Gly-Tyr-NH_2_) was obtained as above reported (4.2.4)^18^.

#### EAK-HVP

The chemoselective ligation between α-ketoaldehyde-7-aminoheptanoic acid-HVP and Aoa-EAK produced the conjugate named EAK-HVP. Details are reported elsewhere (paragraph 3.2.5 and in Zamuner *et al*.^18^). The analytical HPLC of purified EAK-HVP is reported in Supplementary Information (Fig. S4).

#### α-Ketoaldehyde-IGF-1

The conversion of IGF-1 in α-ketoaldehyde-IGF-1 was obtained by addition of 10 mg of IGF-1 (1.307 × 10^−6^ moles) to 26.3 mL of 100 mM pyridoxal-5-phosphate (PLP) in 25 mM sodium phosphate buffer pH 6.5 (2.615 × 10^−3^ moles PLP; IGF-1:PLP = 1:2000) for 3 hrs at 37 °C and 1 hr at room temperature. The characterization of the product is reported elsewhere^18^.

#### EAK-IGF-1

The chemoselective ligation between α-ketoaldehyde-IGF-1 and Aoa-EAK produced the conjugate reported as EAK-IGF-1^18^. The analytical HPLC chromatogram of purified conjugate is reported in Supplementary Information (Fig. S5). Purified EAK-IGF-1 was used for AFM and biological characterization but CD evaluation was not possible due to the exiguity of the material.

#### EAK-RGD

The peptide EAK-RGD (sequence: H-Arg-Gly-Asp-Ala-Glu-Ala-Glu-Ala-Lys-Ala-Lys-Ala-Glu-Ala-Glu-Ala-Lys-Ala-Lys-NH_2_) was synthesized through solid phare peptide synthesis using Rink Amide MBHA resin (0.7 mmol/g; scale 0.125 mmoles) and a fully automated peptide synthesizer (Syro I, Multisynthec, Witten, Germany). The side chain protection employed were: Arg, Pbf; Asp and Glu, OBut; and Lys, Boc. All the couplings were double. After the Fmoc deprotection the resin was washed with DCM and vacuum dried for 1 hr. The peptide was cleaved from the solid support with contemporary side-chain deprotection using the following mixture: 0.125 mL H_2_O MilliQ, 0.125 mL TES, and 4.750 mL TFA (90 min, under magnetic stirring). After cleavage, the resin was filtered, the reaction mixture was concentrated, and the crude peptide precipitated with cold ethyl ether. The identity of crude peptide was ascertained by mass (exp. mass = 1943.06 Da; theor. mass = 1943.16 Da, ESI-TOF). The chromatogram of purified peptide was carried out in the following conditions: column, Symmetry Shield C_8_ (5 µm, 100 Å, 4.6 × 250 mm, Waters); injection volume, 30 µL of 1 mg/mL peptide solution; flow rate, 1 mL/min; eluent A, 0.05% TFA in water; eluent B, 0.05% TFA in CH_3_CN; gradient, from 10%B to 25%B in 30 min, detection at 214 nm. Purity grade 99.9%. The retention time was 15.8 min (Supplementary Information, Fig. S6). The ^1^H NMR spectrum is reported in Fig. S10 of Supplementary Information.

#### EAK-RGD + IKVAV

The peptide EAK-RGD + IKVAV (sequence: H-Arg-Gly-Asp-Ala-Glu-Ala-Glu-Ala-Lys-Ala-Lys-Ala-Glu-Ala-Glu-Ala-Lys-Ala-Lys-Ile-Lys-Val-Ala-Val-NH_2_) was synthesized by standard Fmoc chemistry using Rink Amide MBHA resin (0.7 mmol/g; scale 0.125 mmoles) and a fully automated peptide synthesizer (Syro I, Multisynthec, Witten, Germany). The side chain protection employed were: Arg, Pmc; Asp and Glu, OBut; and Lys, Boc. All the couplings were double. After the Fmoc deprotection the resin was washed with DCM and vacuum dried for 1 hr. The peptide was cleaved from the solid support with contemporary side-chain deprotection using the following mixture: 0.125 mL H_2_O MilliQ, 0.125 mL TES, and 4.750 mL TFA (90 min, under magnetic stirring). After cleavage, the resin was filtered, the reaction mixture was concentrated, and the crude peptide precipitated with cold ethyl ether. After RP-HPLC purification, the identity of crude peptide was ascertained by mass (exp. mass = 2453.40 Da; theor. mass = 2453.81 Da, ESI-TOF). The chromatogram of purified peptide was carried out in the following conditions: column, Symmetry Shield C_8_ (5 µm, 100 Å, 4.6 × 250 mm, Waters); injection volume, 30 µL of 1 mg/mL peptide solution; flow rate, 1 mL/min; eluent A, 0.05% TFA in water; eluent B, 0.05% TFA in CH_3_CN; gradient, from 15%B to 25%B in 20 min, detection at 214 nm. Purity grade 96%. The retention time was 12.5 min (Supplementary Information, Fig. S7). ^1^H NMR analysis of purified EAK-RGD + IKVAV is reported in Supplementary Information, Fig. S11.

#### EAbuK

The peptide EAbuK (sequence: H-Abu-Glu-Abu-Glu-Abu-Lys-Abu-Lys-Abu-Glu-Abu-Glu-Abu-Lys-Abu-Lys-NH_2_; Abu stands for α-aminobutyric acid) was synthesized by standard Fmoc chemistry using Rink Amide MBHA resin (0.72 mmol/g; scale 0.125 mmoles) and a fully automated peptide synthesizer (Syro I, Multisynthec, Witten, Germany). The side chain protection employed were: Glu, OBut and Lys, Boc. The loading of the resin with the first amino acid was carried out with a double coupling, the following four couplings were performed once and all the remaining were double. After the Fmoc deprotection the resin was washed with DCM and vacuum dried for 1 hr. The peptide was cleaved from the solid support with contemporary side-chain deprotection using the following mixture: 0.125 mL H_2_O MilliQ, 0.125 mL TES, and 4.750 mL TFA (90 min, under magnetic stirring). After cleavage, the resin was filtered, the reaction mixture concentrated, and the crude peptide precipitated with cold ethyl ether. The identity of crude peptide was ascertained by mass (exp. mass = 1726.99 Da; theor. mass = 1727.05 Da, ESI-TOF). The chromatogram of purified peptide was carried out in the following conditions: column, Symmetry Shield C_8_ (5 µm, 100 Å, 4.6 × 250 mm, Waters); injection volume, 100 µL of 1 mg/mL peptide solution; flow rate, 1 mL/min; eluent A, 0.05% TFA in water; eluent B, 0.05% TFA in CH_3_CN; gradient, from 12%B to 22%B in 20 min, detection at 214 nm. Purity grade 95%. The retention time was 15.0 min (Supplementary Information, Fig. S8). The ^1^H NMR spectrum is reported in Fig. S12 of Supplementary Information.

### Circular Dicroism analysis

CD spectra were performed at room temperature (25 °C) using a Jasco model J-1500 automatic recording circular dichrograph equipped with Spectra Manager^TM^ software. Cylindrical fused quartz cells of 0.5 mm path length were used. The CD instrument was standardized with D-10 camphorsulphonic acid ammonium salt. Samples were prepared by dissolving weighed quantities of each peptide in a minimum amount of MilliQ water then by adding 10 mM phosphate buffer pH 7 up to a final content of 98% (v/v). Peptide concentration used for CD analysis was 5 × 10^−5^ M.

### Nuclear magnetic resonance spectroscopy (NMR)

NMR characterizations of EAbuK, EAK-RGD, EAK-RGD + IKVAV, and EAK-(GRGDSP)_4_ were performed by using samples about 1 mM in H_2_O/D_2_O (90:10, v-v) at pH values in the range 4–5. NMR experiments were recorded at 298 K on a Varian INOVA 600 MHz spectrometer equipped with a cold probe and Z-gradients. ^1^H mono-dimensional spectra are referred to internal sodium 3-(trimethylsilyl) propionate 2,2,3,3-d4 (TSP). The water resonance was suppressed by using gradients.

### Atomic force Microscopy (AFM)

#### Sample preparation

A solution 0.15% (w/v) of self-assembling peptide in buffer solution was prepared and 3μl was deposited on a freshly cleaned mica surface. After one hour of incubation the samples were gently washed with buffer solution and dried in air.

Samples of EAK-RGD and EAK-RGD + IKVAV correspond to EAK hydrogel enriched with EAK-RGD or EAK-RGD + IKVAV in a ratio EAK: EAK-bioactive molecule of 1: 1, keeping a final hydrogel concentration of 0.15% w/v. For an easier representation scaffolds of EAK/EAK-RGD and EAK/EAK-RGD + IKVAV were named simply EAK-RGD and EAK-RGD + IKVAV.

#### AFM measurements

AFM measurements were carried out with the “J scanner” in tapping mode by using a Nanoscope IIIA-MultiMode AFM (Digital Instruments, Santa Barbara, CA, USA) under room conditions. The force was maintained at the lowest value as possible by continuous adjusting the set point during imaging. Images were recorded using 0.5–2 Ω·cm phosphorous (n) doped silicon tips mounted on cantilevers with a nominal force constant of 40 N/m, a resonance frequency of 300 kHz and a tip curvature radius of 10 nm.

### Biological assays

#### Scaffold preparation

Concentrated SAP solutions (1% w/v) were prepared in deionized water. The final concentration of SAP (0.15% w/v) was obtained adding PBS. The biological evaluation was carried out on EAK hydrogel enriched with EAK-RGD or EAK-RGD + IKVAV in a ratio EAK: EAK-bioactive molecule of 1: 1. For an easier representation scaffolds of EAK/EAK-RGD and EAK/EAK-RGD + IKVAV were named simply EAK-RGD and EAK-RGD + IKVAV. Samples of EAK enriched with conjugates obtained through chemoselective ligation (Fig. 2): EAK-(GRGDSP)_4_, EAK-HVP and EAK-IGF-1, were prepared at the following concentrations: 3.9 × 10^−5^ M of EAK-(GRGDSP)_4_, 5.4 × 10^−5^ M of EAK-HVP and 1.7 × 10^−5^ M of EAK-IGF-1. In a parallel way, EAK/EAK-(GRGDSP)_4_, EAK/EAK-HVP and EAK/EAK-IGF-1 were conveniently named EAK-(GRGDSP)_4_, EAK-HVP and EAK-IGF-1 respectively. All EAK scaffolds enriched with EAK bonded to bioactive sequences were prepared keeping a final hydrogel concentration of 0.15% w/v.

#### Culture of myenteric neurons

Intestinal neurons were isolated from the gut of adult mice^42^. The small intestine was aseptically removed, washed in Hanks balanced salt solution (HBSS; Gibco, Monza, Italy) and cut into pieces of 1 cm length. Intestinal segments were streatched on a sterile glass rod and layers of longitudinal muscle containing the myenteric plexus (LMMP) were obtained. LMMP strips were rinsed twice in HBSS, minced with scissors and dissociated by incubation for 10 min at 37 °C with collagenase type II (1.4 mg/mL, WorthingtonBiochemical Corp, Lakewood, NJ) and DNase (6.25 μg/mL, Calbiochem, Milan, Italy). Following enzymatic inactivation, tissues were further triturated using a pipette and collected by centrifugation (350 x g for 8 min). Cells were then plated on coverslips coated with different SAP scaffolds and incubated in Euromed-N medium (Euroclone, Milan, Italy) supplemented with heat inactivated fetal bovine serum (1% vol/vol), penicillin (100 U mL^−1^), streptomycin (50 μg mL^−1^), and nerve growth factor (10 ng mL^−1^), all purchased from Gibco. Medium was changed 18 hrs after isolation and then replaced every 2 days. Cells were used at the 6^th^ day in culture.

#### Immunocytochemistry

Cultured neurons were washed and fixed in PFA (4% w/vol) for 20 min at room temperature. Cells were then permeabilized with 0.5% Triton X-100 in PBS for 15 min and incubated at room temperature for 1 h with rabbit polyclonal anti-peripherin antibody (Millipore, Milan, Italy). Following extensive washes in PBS, cells were incubated for 1 h at room temperature with TRITC labeled secondary antibodies (Molecular Probe, Milan, Italy). Nuclei were stained with TOTO-3 iodide (Molecular Probe). Samples were then analysed using Leica TCSNT/SP2 confocal microscope. All microscope parameters were set to collect images below saturation and were kept constant during acquisition.

Number of peripherin positive cells was recorded within a minimum of 3 randomly selected fields covering 1.254 mm^2^ from five independent experiments. The percentage of peripherin positive cells was calculated on the number of total nuclei stained with TOTO-3 iodide. Axonal length was measured in stained neuronal cells by using ImageJ 1.40 g (National Institutes of Health; Bethesda, MD, USA). For each experimental condition, 10 cells in 8 images obtained from at least three separate staining sessions were considered. Quantitative analysis was performed manually by using a counting grid, as previously reported^43^.

#### RNA isolation and quantitative PCR

Total RNA was isolated from neurons cultured on different hydrogels using SV Total RNA Isolation System kit (Promega, Milan, Italy) as previously described^44^. Contaminating DNA was removed by DNase I treatment (Promega). cDNA synthesis and subsequent amplification was performed in a one-step using the iTaq Universal SYBR Green One-Step Kit (Bio-Rad). The reaction mixture contained 200 nM forward primer, 200 nM reverse primer, iTaq universal SyBR Green reaction mix, iScript reverse transcriptase, and 200 ng total RNA. Real time PCR was performed using ABI PRISM 7700 Sequence Detection System (Applied Biosystems,Monza, Italy). Expression of Glyceraldehyde 3-phosphate dehydrogenase (Gapdh) was used as internal control. Experiments were performed in duplicate from three independent samples. The mean normalized fold expression ± SEM are plotted. Oligonucleotides used are listed in Table 1.

**Table 1:** Oligonucleotides used for quantitative PCR analysis. Fw: forward; Rv: reverse.

Gene [accession #]	Sequence
Gapdh [NM_001289726]	Fw 5′-AGTGCCAGCCTCGTCCCGTA-3′
	Rv 5′-CAGGCGCCCAATACGGCCAA-3′
Ntf3 [NM_001164034]	Fw 5′-CGACGTCCCTGGAAATAGTC-3′
	Rv 5′-TGGACATCACCTTGTTCCACC-3′
Ngf [NM_001112698]	Fw 5′-AGTTTTGGCCTGTGGTCGT-3′
	Rv 5′-GGACATTACGCTATGCACCTC-3′
Ache [NM_009599]	Fw 5′-CTTTCTCCCCAAATTGCTCA-3′
	Rv 5′-TTCCAGTGCACCATGTAGGA-3′
Chat [NM_009891]	Fw 5′-GGTTCGGTGCGTAACAGC-3′
	RV 5′-GCGATTCTTAATCCAGAGTA-3′

#### Statistical analysis

Biological data are presented as the mean ± standard error of the mean (SEM). Differences in the mean beween experimental groups were tested using Student’s t-test or one-way ANOVA analysis followed by Bonferroni multicomparison post hoc test. A p value of 0.05 or less was considered statistical significant. Statistical analyses were performed using GraphPad Prism 3.03 software (GraphPad, San Diego, CA).

### Experimental Protocols

Animal experiments were approved by the Animal Care and Use Committee of the University of Padova under license from the Italian Ministry of Health (244/2015-PR). All animal procedures were carried out in accordance with the National and European guidelines for handling and use of experimental animals.

## Supplementary information

Supplemetary Information
