# Supplementary material for: 3D Synthetic Peptide-based Architectures for the Engineering of the Enteric Nervous System
Source: Sci Rep. 2019 Apr 3;9:5583. doi: 10.1038/s41598-019-42071-7 (PMC6447567; doi:10.1038/s41598-019-42071-7)
Supplement: Supplementary file 1 — Supplemetary Information [file 41598_2019_42071_MOESM1_ESM.docx]

**3D Synthetic Peptide-based Architectures for the Engineering of the Enteric Nervous System**

**Paola Brun^a^, Annj Zamuner^b^, Alessandro Peretti^b^, Jessica Conti^a^, Grazia M.L. Messina^c^, Giovanni Marletta^c^, Monica Dettin^b*^**

^a^Department of Molecular Medicine, University of Padova, Via Gabelli, 63, Padova, 35121, Italy

^b^Department of Industrial Engineering, University of Padova, Via Marzolo, 9, Padova, 35131, Italy

^c^Department of Chemical Sciences, University of Catania, Via A. Doria, 6, Catania, 95125, Italy

*corresponding author, monica.dettin@unipd.it

**Supplementary Information**

**CD analysis**

**Figure S1.** CD spectra of HVP. The CD analysis was carried out in 10 mM phosphate buffer pH 7 with a peptide concentration of 1.9028 × 10^-5^M.

**HPLC analysis:**

******

**Figure S2.** Analytical HPLC chromatogram of EAK. Column: Jupiter C_18_ (5μm, 300 Å, 4.6×250 mm); injection:100 µL; flow: 1 mL/min; eluent A: 0.05% TFA in H_2_O MilliQ; eluent B: 0.05% TFA in CH_3_CN; gradient: from 10% to 20% of eluent B in 20 min; λ=214 nm; time of retention: 10.24 min.

**Figure S3.** Analytical HPLC chromatogram of EAK-(GRGDSP)_4_. Column: Jupiter C_18_ (5 μm, 300 Å, 4.6 x 250 mm); flow: 1 mL/min; eluent A: 0.05% TFA in H_2_O MilliQ; eluent B: 0.05% TFA in CH_3_CN; gradient: from 0 to 35 % of eluent B in 35 min, λ=214 nm; time of retention: 21.71 min.

**Figure S4.** Analytical HPLC chromatogram of EAK-HVP. Column: Jupiter C_18_ (5 μm, 300 Å, 4.6 x 250 mm); flow: 1 mL/min; eluent A: 0.05% TFA in H_2_O MilliQ; eluent B: 0.05% TFA in CH_3_CN; gradient: from 10 to 30 % of eluent B in 20 minutes, λ=214 nm; time of retention: 14.81 min.

**Figure S5.** Analytical HPLC chromatogram of EAK-IGF-1. Column: Jupiter C_18_ (5 μm, 300 Å, 4.6 x 250 mm); flow: 1 mL/min; eluent A: 0.05% TFA in H_2_O MilliQ; eluent B: 0.05% TFA in CH_3_CN; gradient: from 10 to 50 % of eluent B in 40 min, λ=214 nm; time of retention: 24.28 min.

**Figure S6.** Analytical HPLC chromatogram of EAK-RGD. Column: Symmetry Shield C_8_ (5μm, 100 Å, 4.6×250 mm); injection: 30 µL; flusso: 1 mL/min; eluent A: 0.05% TFA in H_2_O MilliQ; eluent B: 0.05% TFA in CH_3_CN; gradient: from 10% to 25% of eluent B in 30 min; λ=214 nm; time of retention: 15.81 min.

*__*

**Figure S7.** Analytical HPLC chromatogram of EAK-RGD+IKVAV. Column: Symmetry Shield C_8_ (5μm, 100 Å, 4.6×250 mm); injection: 30 µL; flow: 1 mL/min; eluent A: 0.05% TFA in H_2_O MilliQ; eluent B: 0.05% TFA in CH_3_CN; gradient: from 15% al 25% of eluent B in 20 min; λ=214 nm; time of retention: 12.55 min.

*__*

**Figure S8.** Analytical HPLC chromatogram of EAbuK. Column: Symmetry Shield C_8_ (5 µm, 100 Å, 4.6 × 250 mm); flow: 1 mL/min; injection: 100 µL; eluent A: 0.05% TFA in H_2_O MilliQ; eluent B: 0.05% TFA in CH_3_CN; gradient: from 12% to 22% of eluent B in 20 min; λ=214 nm; time of retention: 15.08 min.

**NMR analysis:**


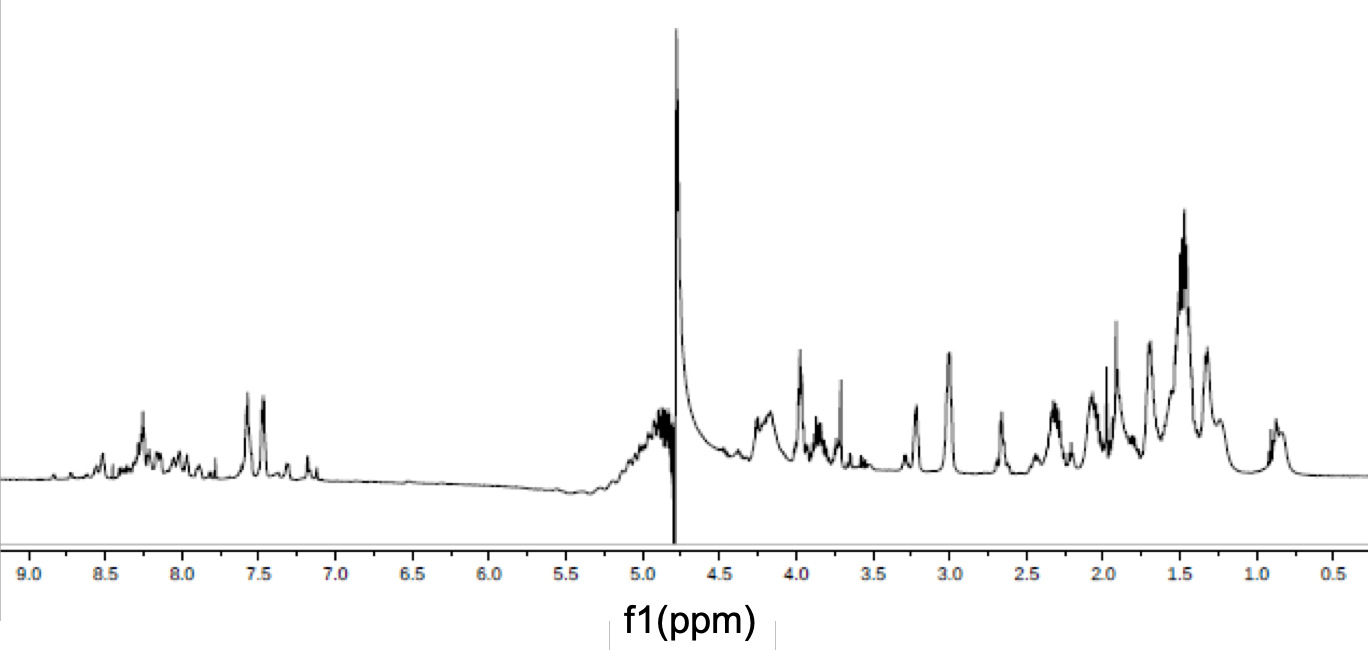


**Figure S9.** ^1^H NMR spectrum of EAK-(GRGDSP)_4_ (600 MHz) in H_2_O/D_2_O (90:10, v:v).


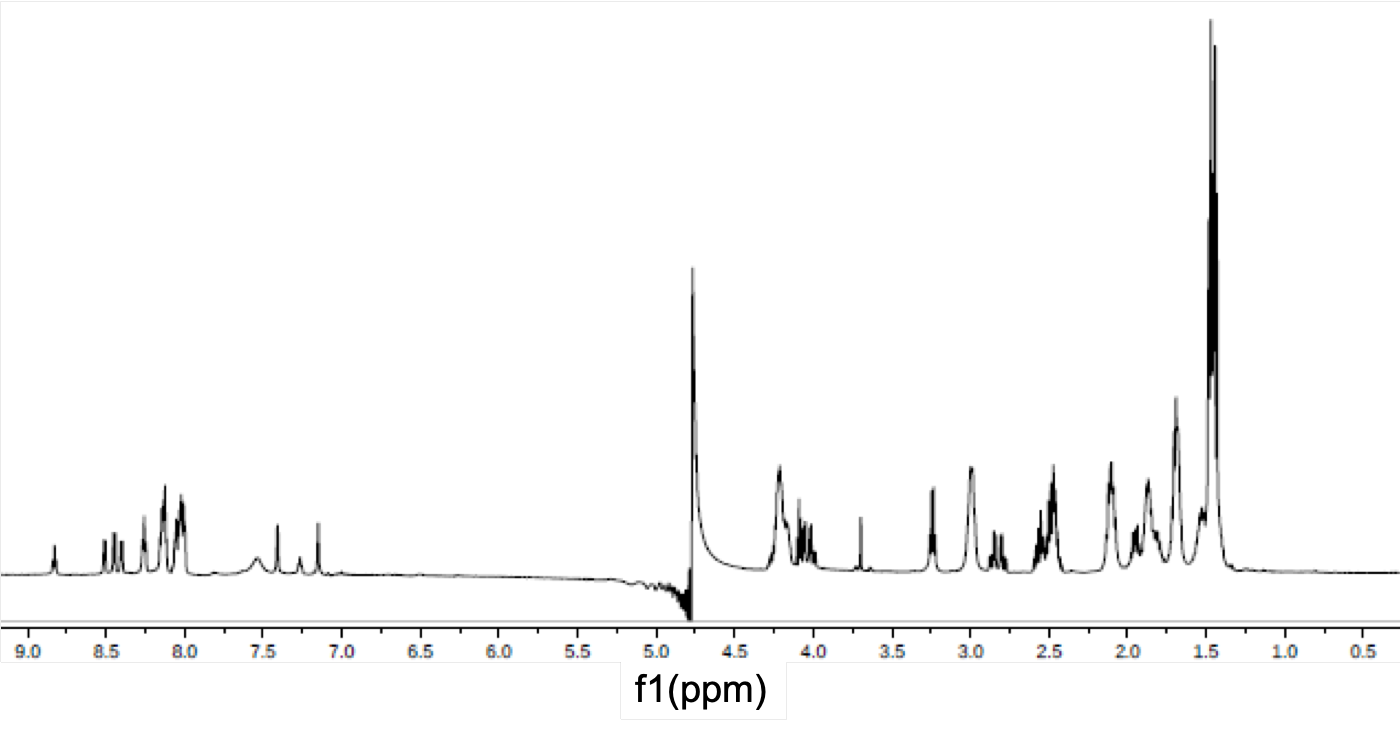


**Figure S10.** ^1^H NMR spectrum of EAK-RGD (600 MHz) in H_2_O/D_2_O (90:10, v:v).

.


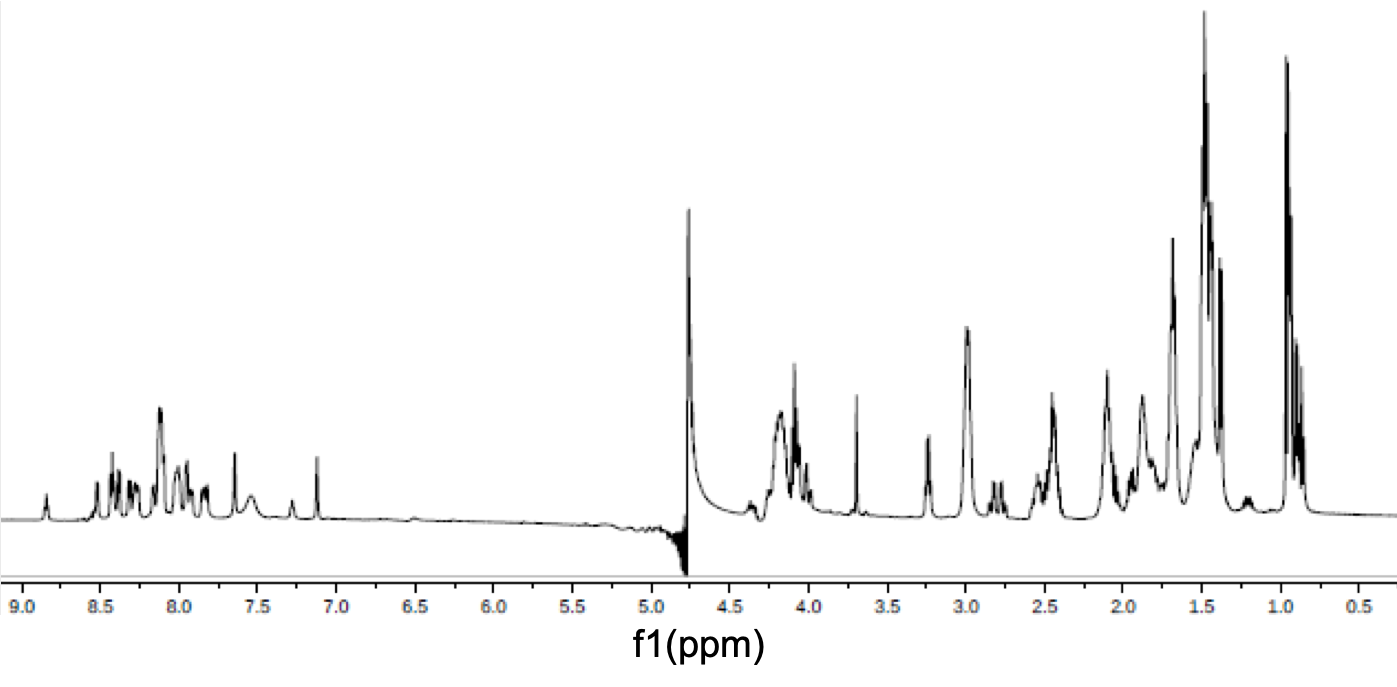


**Figure S11.** ^1^H NMR spectrum of EAK-RGD+IKVAV (600 MHz) in H_2_O/D_2_O (90:10, v:v).


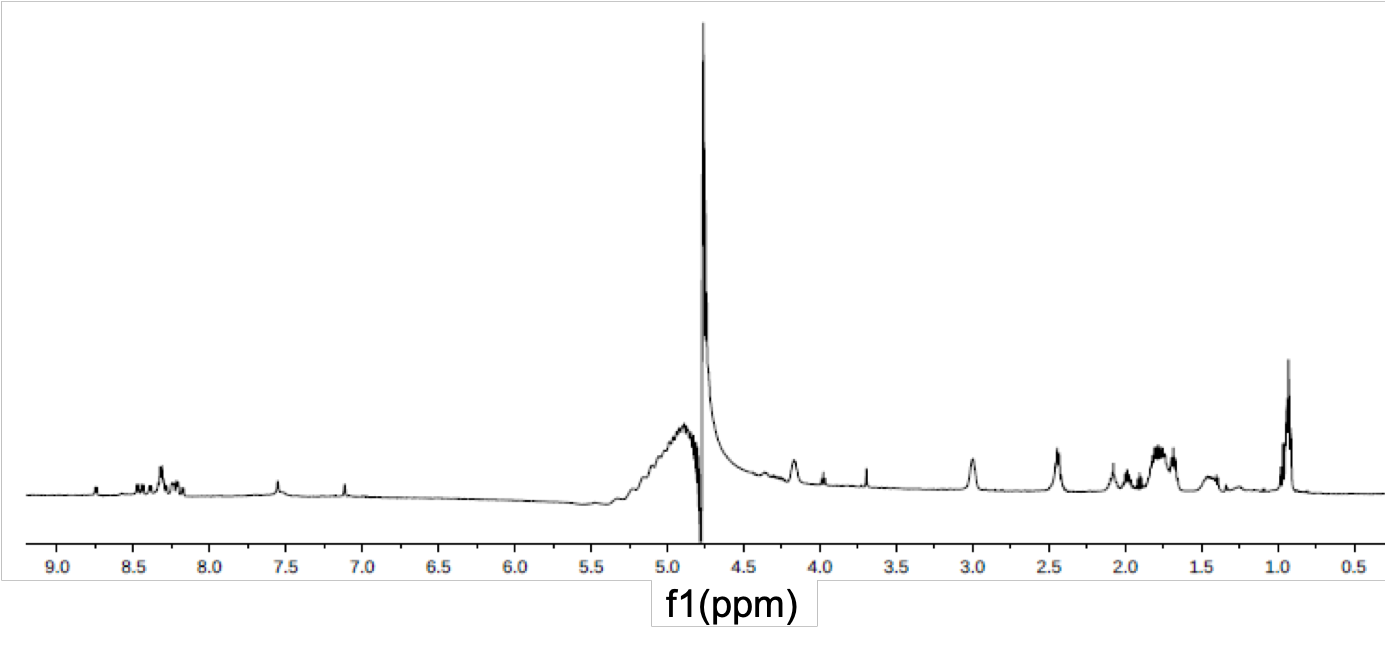


**Figure S12.** ^1^H NMR spectrum of EAbuK (600 MHz) in H_2_O/D_2_O (90:10, v:v).
